# Flap Sliding Technique for Managing Flap Striae following Laser In Situ Keratomileusis

**DOI:** 10.1155/2020/5614327

**Published:** 2020-02-24

**Authors:** Khaled Abdelazeem, Mohamed A. Nassr, Hazem Abdelmotaal, Ehab Wasfi, Dalia Mohamed El-Sebaity

**Affiliations:** ^1^Department of Ophthalmology, Faculty of Medicine, Assiut University, Assiut, Egypt; ^2^Alforsan Eye Center, Assiut, Egypt; ^3^North West Anglia NHS Foundation Trust, Peterborough, UK

## Abstract

**Purpose:**

To assess the efficacy and safety of a simple, noninvasive, “flap-sliding” technique for managing flap striae following laser in situ keratomileusis (LASIK).

**Methods:**

This prospective, interventional study included eyes with post-LASIK flap striae. All eyes underwent flap sliding 1-2 days after surgery. Following flap edge epithelialisation, a cellulose sponge was used to gently slide the flap perpendicular to the striae direction. This technique allows for flap striae treatment without flap lifting, avoiding any associated lifting complications. Uncorrected distance visual acuity (UDVA), corrected distance visual acuity (CDVA), and refractive error were monitored one day after the flap-sliding procedure.

**Results:**

Fifteen eyes (15 patients) with post-LASIK flap striae were managed using the flap-sliding technique. The procedure did not successfully relocate the flap striae in 1 eye, and flap elevation and floating (using a balanced salt solution) were required. Therefore, 14 eyes were included in post-flap-sliding analyses. The UDVA improved in all patients the first day after the flap-sliding procedure was performed, with 11 of 14 eyes (78.57%) reaching an UDVA of 20/25 or better. Complications following flap sliding occurred in 2 eyes (14.29%). One eye had intraoperative epithelial abrasion, and 1 eye had residual postoperative striae outside of the optical zone.

**Conclusion:**

The flap-sliding technique is a simple, noninvasive, efficient, and safe technique for managing post-LASIK flap striae that develop after epithelial healing in the early post-LASIK period. This trial is registered with NCT04055337.

## 1. Introduction

Laser in situ keratomileusis (LASIK) is currently the most commonly performed corneal refractive surgery [1]. Although outcomes are highly predictable, successful, and satisfactory, intraoperative and/or postoperative complications can occur. Most complications are flap related and include a free cap, a button hole, an incomplete cut, flap striae, interface debris, diffuse lamellar keratitis (DLK), and epithelial ingrowth [2, 3]. Flap striae can result in a significant decrease in corrected distance visual acuity (CDVA) and cause glare symptoms. Flap striae may develop from corneal flap misalignment, postoperative flap movement, or a size difference between the flap and the ablated stromal bed. This size difference occurs more often when correcting large refractive errors (“tenting” effect [4, 5]), even after flap creation with a femtosecond laser [6]. Excessively thin or thick flaps, irregular flaps, or free caps are associated with a higher incidence of post-LASIK flap striae. Therefore, the LASIK flap should be accurately aligned in the stromal bed, with symmetrical gutters around the flap, to minimize flap striae incidence [7, 8].

Various techniques have been used to treat flap striae, but early detection is crucial. Lifting and refloating the flap within the first 24 hours of LASIK will likely result in striae resolution [9]. The full range of treatment modalities includes refloating, stretching and smoothing, hypotonic saline irrigation, flap massage, hyperthermia, the sandwich compression maneuver, epithelium removal, Bowman's layer phototherapeutic keratectomy (PTK), and flap suturing. These techniques vary from noninvasive to highly invasive and are chosen based on striae severity and duration [4, 5, 7, 8, 10–12]. This study examined the efficacy and safety of a simple, noninvasive, “flap-sliding” technique performed within 2 days of LASIK to treat post-LASIK flap striae.

## 2. Materials and Methods

### 2.1. Study Design and Subjects

This prospective, interventional study was reviewed and approved by the Assiut University Institutional Review Board. All study conducted adhered to the tenets of the Declaration of Helsinki. All subjects provided written informed consent to participate in the study, following a discussion about the nature of the study and the risks/benefits of participation.

This study included eyes that developed flap striae in the visual zone after microkeratome LASIK. The single-use Moria M2 microkeratome (Moria, Antony, France) was used to create flaps. The same blade was used sequentially for both eyes in bilateral cases starting with the right eye. Striae were identified within 2 days of surgery and significantly interfered with vision. Flap striae were considered to be visually significant if CDVA was at least 1 line worse than pre-LASIK CDVA, and there was a significant postoperative refractive error and/or halo or glare were present. Subjects were excluded from participation if they exhibited striae 2 days after surgery or exhibited visually insignificant striae. Data collected included subject age and pre-LASIK minimum corneal thickness, anterior corneal curvature, and spherical equivalent (SE) of the refractive error. After LASIK (before flap sliding) data collected included uncorrected distance visual acuity (UDVA) and SE of the refractive error. One day after flap sliding, UDVA and SE of the refractive error were reexamined. A decimal visual acuity (VA) chart was used to measure VA. A WaveLight Oculyzer II (Alcon Surgical, USA) equipped with a Scheimpflug camera was also used to measure LASIK flap thickness and corneal curvature.

### 2.2. Surgical Technique

All subjects underwent the “flap-sliding” technique, which was performed at Alforsan Eye Centre in Assiut, Egypt. Prior to surgery, all subjects underwent corneal imaging with anterior segment optical coherence tomography (OCT; Triton-DRI swept source OCT, Topcon Co., Tokyo, Japan) to confirm the presence of striae. All surgical procedures were performed by the same surgeon (KA) 1 or 2 days following LASIK with the aid of a slit lamp mounted on the microscope of the Eye-Q system (WaveLight Allegretto Wave Eye-Q 400 Hz, Alcon Laboratories, Inc. Fort Worth, TX).

**Figure 1:**
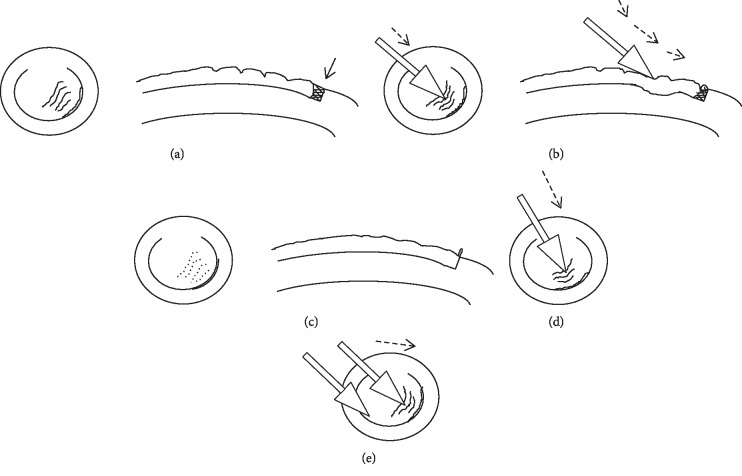
Steps of the flap-sliding procedure. (a) Identify flap striae direction. (b) Use a partially wetted cellulose sponge to gently slide the flap in a direction perpendicular to the striae. (c) Watch for the epithelial fold sign at the flap edge to indicate that the flap is in its proper position. This fold represents the epithelium that grew over the bed and is pushed away. When properly seated, the epithelium at the flap edge covers the exposed bed (arrow, (a)). Direction of flap sliding for horizontal (d) and vertical (e) striae.

Subjects were administered a topical anesthetic (benoxinate hydrochloride 0.4%, Egyptian International Pharmaceutical Industries, Co., 10^th^ of Ramadan City, Egypt). Striae location and orientation were then determined (Figure 1(a)) after drying the corneal surface with a cellulose sponge. The partially hydrated sponge (used to prevent epithelium injury during flap sliding) was used to push the LASIK flap over the stromal bed in a direction that was perpendicular to the striae. The flap was pushed from the proximal side of the striae toward the flap edge (Figure 1(b)). For horizontally oriented striae, the flap hinge supported the flap against the push (Figure 1(d)). However, for vertically oriented striae, two sponges were needed. One sponge supported the flap near the edge, which was supposed to be in its normal place, and one sponge pushed the flap toward the opposite flap edge (Figure 1(e)). The ultimate goal was to move striae and put the flap into its proper place. This was indicated by the appearance of a continuous, crescent-shaped epithelial fold that was oriented parallel to striae, located at the flap edge, and created in the direction of flap sliding (Figure 1(c)). This fold represents the epithelium that grew over the bed and is pushed away. Following surgery, OCT imaging was repeated. This technique is demonstrated in [Supplementary-material supplementary-material-1].

### 2.3. Data Analysis

Data are presented as mean ± standard deviation. Mean and standard deviations were calculated using Microsoft Excel 2010 (Microsoft Corporation, Redmond, WA). Paired *t*-tests were used to examine differences between continuous variables. The *p* values were calculated using GraphPad Prism (version 6.01, GraphPad Software, La Jolla, CA). Statistical significance was defined as *p* < 0.05.

## 3. Results

Fourteen eyes (4 right eyes, 10 left eyes) of 14 patients (5 men, 9 women) with LASIK flap striae were included in this study. The mean subject age was 28.43 ± 6.88 years. Before LASIK, the anterior corneal surface *K*-value was 44.08 ± 1.21 diopters (D), minimum corneal thickness was 525.57 ± 25.76 *μ*m, and SE was −5.54 ± 2.59 D. Additionally, mean CDVA was 0.99 ± 0.04 (Snellen equivalent: 20/20). Mean LASIK flap thickness was 111.43 ± 5.35 *μ*m.

**Figure 2:**
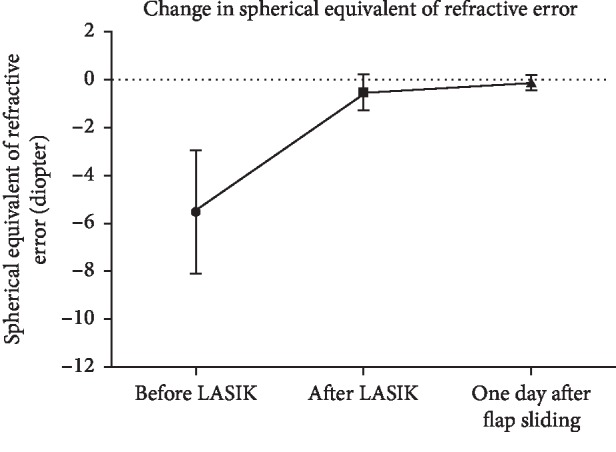
Changes in the refractive error before LASIK, after LASIK, and one day after flap sliding.

**Figure 3:**
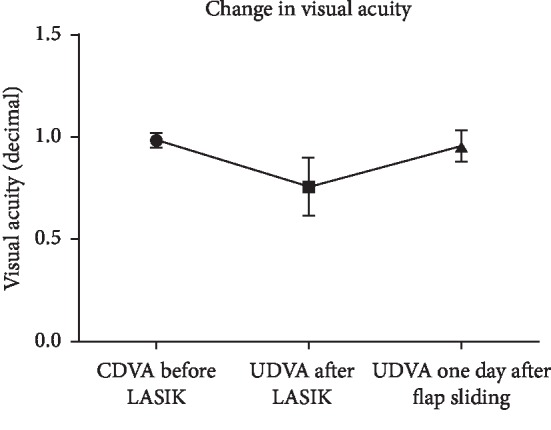
Changes in visual acuity before LASIK, after LASIK, and one day after flap sliding. CDVA, corrected distance visual acuity; UDVA, uncorrected distance visual acuity.

**Figure 4:**
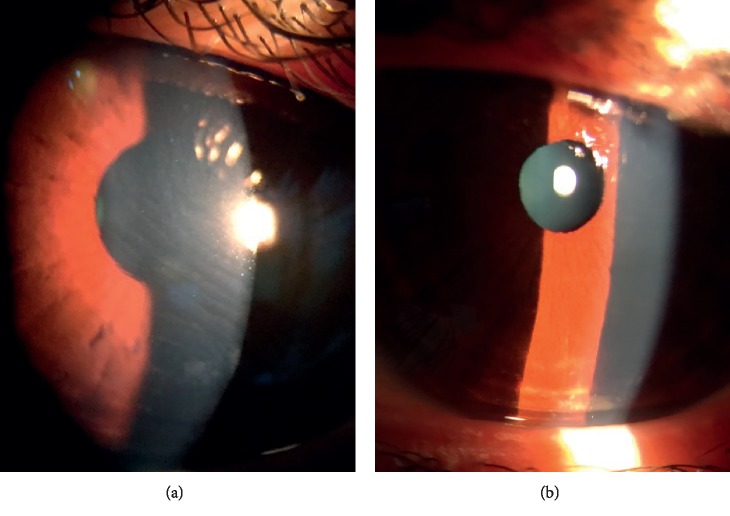
Representative color slit-lamp photographs of a 22-year-old woman who developed flap striae following LASIK. Photographs were taken before (a) and one day after (b) the subject underwent flap-sliding procedure.

**Figure 5:**
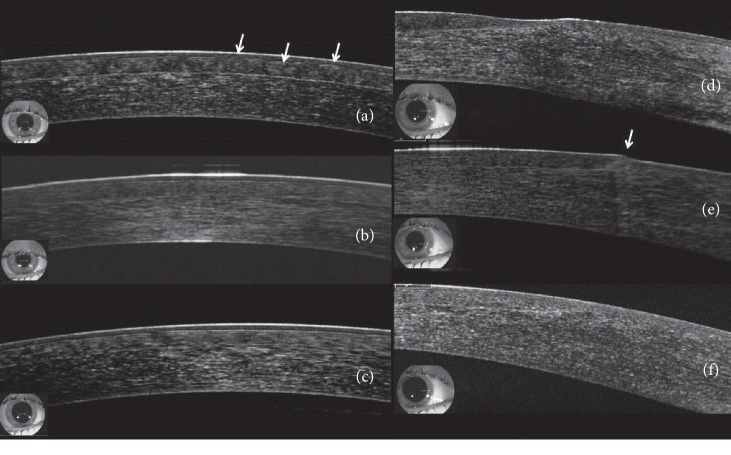
Anterior segment optical coherence tomography (AS-OCT) scans of a 24-year-old woman who developed flap striae following LASIK. (a) Images obtained before flap sliding show dome-shaped [13] irregularities of the flap (arrows). (b) One hour after undergoing the flap-sliding procedure, the dome-shaped irregularities have improved and now appear as fine corrugations on the flap surface. (c) Two days after undergoing flap sliding, flap striae have completely resolved and the corneal surface appears smooth. (d) Images of the flap edge obtained before flap siding show gapping between the flap edge and the exposed stromal bed. (e) One hour after flap sliding, flap gapping is no longer apparent and epithelial cells have aggregated at the flap edge (arrow). (f) Two days after flap sliding, the flap is perfectly aligned and no epithelial irregularities at the flap edge are visible.

Mean SE decreased to −0.54 ± 0.75 D following LASIK, but UDVA was 0.76 ± 0.14 (20/26), which was significantly worse than pre-LASIK CDVA (*p* < 0.0001). One day after flap sliding was performed, mean UDVA significantly improved to 0.96 ± 0.08 (20/21, *p*=0.001) and SE significantly decreased to −0.12 ± 0.29 D (*p*=0.001). Following flap sliding, UCDA was not significantly different from pre-LASIK CDVA (*p*=0.1039). All subjects had an improvement in UDVA after undergoing flap sliding, with 11 of 14 subjects (78.57%) attaining pre-LASIK CDVA 1 day after flap sliding (Figures 2 and 3). Furthermore, 13 of 14 subjects (92.86%) had complete resolution of flap striae 1 day after undergoing the flap sliding procedure (Figure 4). One subject had residual peripheral striae outside of the optical zone. Furthermore, anterior segment OCT performed immediately and 2 days after flap sliding demonstrated striae resolution and proper flap edge alignment (Figure 5).

Complications of this procedure have been recorded in 2 cases (14.29%) as follows. Intraoperative epithelial abrasion occurred in 1 case. The other case had residual postoperative striae outside of the optical zone. Additionally, striae were not adequately repositioned in 1 eye, which subsequently underwent flap elevation and floating with a balanced salt solution (BSS) immediately following flap sliding. Post-flap sliding data from this case were excluded from analyses.

## 4. Discussion

The flap-sliding technique is an effective method for managing flap striae after LASIK. The UDVA improved in all subjects, with the majority (78.57%) reaching their pre-LASIK CDVA 1 day after undergoing flap sliding. Additionally, striae completely resolved following flap sliding in 13 of 14 eyes (91.86%). The 1 eye without striae resolution had residual striae outside of the optical zone that were not visually significant. Epithelial abrasion occurred in one eye because of a relatively dry cellulose sponge. This simple technique was based on the fact that only weak flap healing occurs at the interface, even several years after LASIK. Even though the flap is in close association with the stromal bed, severed lamellae do not reconnect. In contrast, healing at the flap margin is much stronger (10 times that of the interface) [14, 15]. However, overall flap healing is weak, as demonstrated by the occurrence of late-onset traumatic flap displacement [16].

The presence of flap striae after LASIK may cause patient dissatisfaction, making intervention important. Some surgeons recommend flap stretching with a cotton-tipped applicator (performed at the slit-lamp biomicroscope), which is a simpler, less invasive approach than flap lifting. However, flap stretching can only be performed during the immediate postoperative period, before the flap edge epithelium has healed [12]. In contrast, later interventions, which are performed after the flap edge epithelium has healed, require more invasive techniques. These procedures carry the risk of infection, epithelial erosion, epithelial ingrowth, and DLK. Additionally, the incidence of epithelial ingrowth is greater after flap relifting than after the primary LASIK procedure [17–22]; in severe cases, epithelial ingrowth can require treatment with lamellar keratoplasty [23]. The appearance of a crescent-shaped epithelial fold at the end of flap sliding is important for two reasons: first, it is an indicator of perfect flap alignment; second, it signifies that the epithelium is not below the repositioned flap, indicating a reduced risk of epithelial ingrowth.

Our study was limited by its relatively small number of subjects and further studies on a larger number of cases are needed. Additionally, the current study only included cases in which flap sliding was performed within 2 days of LASIK. Therefore, further study is needed on eyes that undergo flap sliding later in the healing process. In conclusion, flap sliding is a simple, noninvasive, efficient, and safe technique for managing post-LASIK flap striae that develop after early post-LASIK epithelium healing. In contrast to flap massage, which displaces striae toward the periphery, the flap-sliding technique eliminates striae from the entire flap. Therefore, the flap-sliding technique should be considered for managing flap striae before turning to more invasive procedures. Additionally, flap sliding can be done in an outpatient setting at a slit-lamp biomicroscope.

## Data Availability

All data files used to support the findings of this study are available from the corresponding author upon request.

## Abbreviations

BSS: Balanced salt solution
CDVA: Corrected distance visual acuity
DLK: Diffuse lamellar keratitis
LASIK: Laser in situ keratomileusis
OCT: Optical coherence tomography
PTK: Phototherapeutic keratectomy
SE: Spherical equivalent
UDVA: Uncorrected distance visual acuity
VA: Visual acuity.

## Supplementary Materials

Supplementary Materials The supplementary file is a short video about “Steps of flap-sliding technique.”
